# SbSI Nanosensors: from Gel to Single Nanowire Devices

**DOI:** 10.1186/s11671-017-1854-x

**Published:** 2017-02-07

**Authors:** Krystian Mistewicz, Marian Nowak, Regina Paszkiewicz, Anthony Guiseppi-Elie

**Affiliations:** 1 Institute of Physics - Center for Science and Education, Silesian University of Technology, Krasińskiego 8, 40-019 Katowice, Poland; 2 Division of Microelectronics and Nanotechnology, Wrocław University of Technology, Wrocław, Poland; 3 Center for Bioelectronics, Biosensors and Biochips, Clemson University, 132 Earle Hall, Clemson, USA

**Keywords:** Nanowires, Gas sensors, Antimony sulfoiodide (SbSI), Humidity, Carbon dioxide

## Abstract

The gas-sensing properties of antimony sulfoiodide (SbSI) nanosensors have been tested for humidity and carbon dioxide in nitrogen. The presented low-power SbSI nanosensors have operated at relatively low temperature and have not required heating system for recovery. Functionality of sonochemically prepared SbSI nanosensors made of xerogel as well as single nanowires has been compared. In the latter case, small amount of SbSI nanowires has been aligned in electric field and bonded ultrasonically to Au microelectrodes. The current and photocurrent responses of SbSI nanosensors have been investigated as function of relative humidity. Mechanism of light-induced desorption of H_2_O from SbSI nanowires’ surface has been discussed. SbSI nanosensors have been tested for concentrations from 51 to 10^6^ ppm of CO_2_ in N_2_, exhibiting a low detection limit of 40(31) ppm. The current response sensitivity has shown a tendency to decrease with increasing CO_2_ concentration. The experimental results have been explained taking into account proton-transfer process and Grotthuss’ chain reaction, as well as electronic theory of adsorption and catalysis on semiconductors.

## Background

Nanoferroelectrics [1–4] have currently been studied with increasing intensity due to their importance for applications in ferroelectric non-volatile random access memory devices and FRAMs and as passive capacitors for volatile dynamic random access memories, DRAMs, electromechanical systems, actuators, energy-harvesting devices, and gas sensors. Antimony sulfoiodide (SbSI) exhibits plenty of outstanding strongly coupled semiconductive and ferroelectric properties [5, 6]. Recently [7–10], sonochemically prepared SbSI nanowires have been demonstrated to be suitable candidates for high-sensitivity gas detection. Due to large surface-to-volume ratio, the reaction between target gas and nanosensor surface can extremely occur [11]. Moreover, dipole moments of gas molecules can interact with electric polarization of some ferroelectric domains at SbSI surface, giving a stronger and more measurable sensor response. It should be underlined that, in the case of semiconductors, the existence of surface layer affected by adsorbed species strongly influences electrical properties of a sensor.

The aim of this paper was to compare functionality of sonochemically prepared SbSI nanosensors made of xerogel as well as single nanowires. The gas-sensing properties of the nanosensors were tested for humidity and carbon dioxide (CO_2_) in nitrogen. To the best of our knowledge, the electrical response of SbSI nanowires affected by CO_2_ adsorption is reported for the first time.

## Methods

### Material Synthesis

SbSI xerogel was prepared sonically from the constituents (the elements: antimony, sulfur, and iodine). The component mixture was immersed at room temperature and ambient pressure in ethanol, which was contained in a polyethylene/polypropylene cylinder. The vessel was closed during the experiment to prevent volatilization of the precipitant in long-time tests. The cylinder was partly submerged in water in cup-horn of ultrasonic reactor (750 W ultrasonic processor VCX-750 with sealed converter VC-334 (Sonics & Materials, Inc.). The used ultrasounds had 20 kHz and 565 W/cm^2^ power density guaranteed by the manufacturer. The cup-horn was filled with water continuously pumped through refrigerated circulating bath AD07R (PolyScience). The sonolysis was carried out at the temperature of 323 K within 2 h. Other details of the used experimental setup and applied procedure were described elsewhere [12, 13]. When the process was finished, the ethanol was evaporated and the so-called SbSI xerogel was obtained.

It was established in [12, 13] from high-resolution transmission electron microscopy, selected area electron diffraction, and powder X-ray diffraction, that SbSI gel, fabricated sonochemically by using the described equipment and procedure, consisted of crystalline nanowires. The nanowires had lateral dimensions in range from 10 to 50 nm and average lengths reaching up to several micrometers.

### Sensor Fabrication

**Fig. 1:**
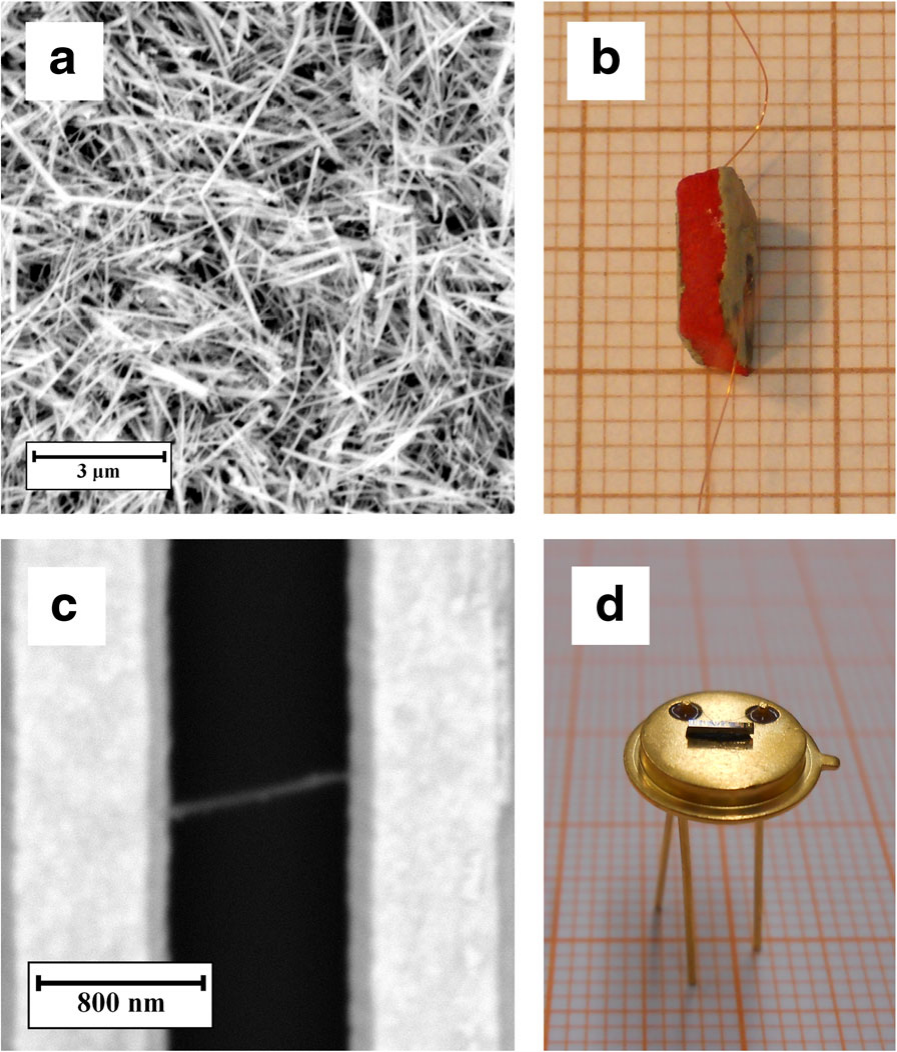
**a** Scanning electron micrograph of a part of sensor (**b**) constructed from sonochemically prepared SbSI xerogel. **c** SEM micrograph of a single nanowire (a part of an array of a few nanowires) aligned in electric field and bonded ultrasonically to Au microelectrodes on Si/SiO_2_ substrate that was integrated in standardized metal semiconductor package TO-5 (**d**)

Two kinds of SbSI nanosensors were constructed. The first one, made as rectangular samples of SbSI xerogel, was cut from the synthesized and dried material. These sensors consisted of large number of chaotically oriented nanowires (Fig. 1a). The dimensions of the sample were 5.70(1) mm × 6.52(1) mm × 3.45(1) mm. The largest opposite surfaces of the sample were covered with a silver paste (SPI Supplies). Electrical connections from these electrodes were made of copper wire (Fig. 1b).

The fabrication of the second type of SbSI nanosensor can be briefly described as follows. In the first step, SbSI xerogel was dispersed in toluene (e.g., in ratio: 0.05 mg SbSI gel/1 ml toluene) using ultrasonic reactor (InterSonic IS-UZP-2). A droplet of dispersed solution was placed onto Si/SiO_2_ substrates or onto glass chips (model IAME-co-IME2-1AU made by Abtech Scientific Inc.) using insulin syringe equipped with 31G needle. These substrates were equipped with gold microelectrodes separated by a gap of 1 μm. The direct current electric field-assisted technique [14] was used to align the nanowires perpendicularly to the electrodes. During the deposition of SbSI sol, electric field of 5 × 10^5^ V/m was applied to electrodes on Si/SiO_2_ substrate. The control of SbSI sol concentration is allowed to obtain an array of a few nanowires. The samples were dried in a glove box 830-ABC/EXP (Plas-Labs Products). In the next step, ultrasonic bonding technique was used to connect SbSI nanowires with Au microelectrodes (Fig. 1c). The detailed description of a setup for ultrasonic processing, applied procedure, and parameters of the process were presented in [7, 9]. The Si/SiO_2_ substrate with array of a few SbSI nanowires was stuck to standardized metal semiconductor package TO-5 (Fig. 1d). The Au microelectrodes were connected with the TO-5 pins using HB05 wire bonder (TPT Wire Bonder). The TO-5 packages were easily mounted in a socket of measurement system. Glass chips IAME-co-IME2-1AU were connected with measurement system using STC 7 Test Clip (Abtech Scientific Inc.).

### Sensor Characterization

Morphology of the fabricated SbSI nanosensors was studied using Phenom Pro X SEM microscope (Phenom World) (Fig. 1a, c). The applied acceleration voltage was 10 kV. The experimental setup for testing the gas sensitivity of SbSI nanosensors had the following components: test chamber equipped with TW70H turbomolecular vacuum pump (Prevac); vacuum gauge controller ACM 1000 with gauges Alcatel ACC 1009, ADS 1001, and ADS 1004; and humidity sensor SHT15 (Sensirion AG) with humidity meter ES-1530 (Elektro-System s.c.). DC electric measurements were performed using Keithley electrometer Model 6517A as well as Keithley 6430 Sub-Femtoamp Remote SourceMeter. Acquisition of the data was realized using PC computer with GPIB bus and appropriate program in LabView (National Instruments). Sample illumination was realized by using Ar laser (*λ* = 488 nm, model Reliant 50s, Laser Physics). The illumination intensity (I_L_) was determined using Keithley 6517A electrometer and Hamamatsu S2387 photodiode in short-circuit regime.

Dry nitrogen with high purity level of 99.999% was chosen as a carrier gas for water vapor and carbon dioxide. Relative humidities (RHs) in range from 0 to 77% were maintained by passing the N_2_ gas to the test chamber over water in special container, whereas CO_2_ concentration (from 51 to 10^6^ ppm) in N_2_ was adjusted using mass flow controllers SLA 5850 (Brooks Instruments) with a real-time monitor computer. A constant temperature of sensors was maintained using thermostat HAAKE DC30 with Kessel HAAKE K20 circulator (Thermo Scientific) and Pt-100 sensor with 211 temperature controller (Lake Shore). Before each experiment, SbSI nanosensors were held at 323 K in vacuum (10^−2^ Pa) for 30 min. Recovery of the sensors was simply realized by evacuation of gas from the test chamber.

## Results and Discussion

**Fig. 2:**
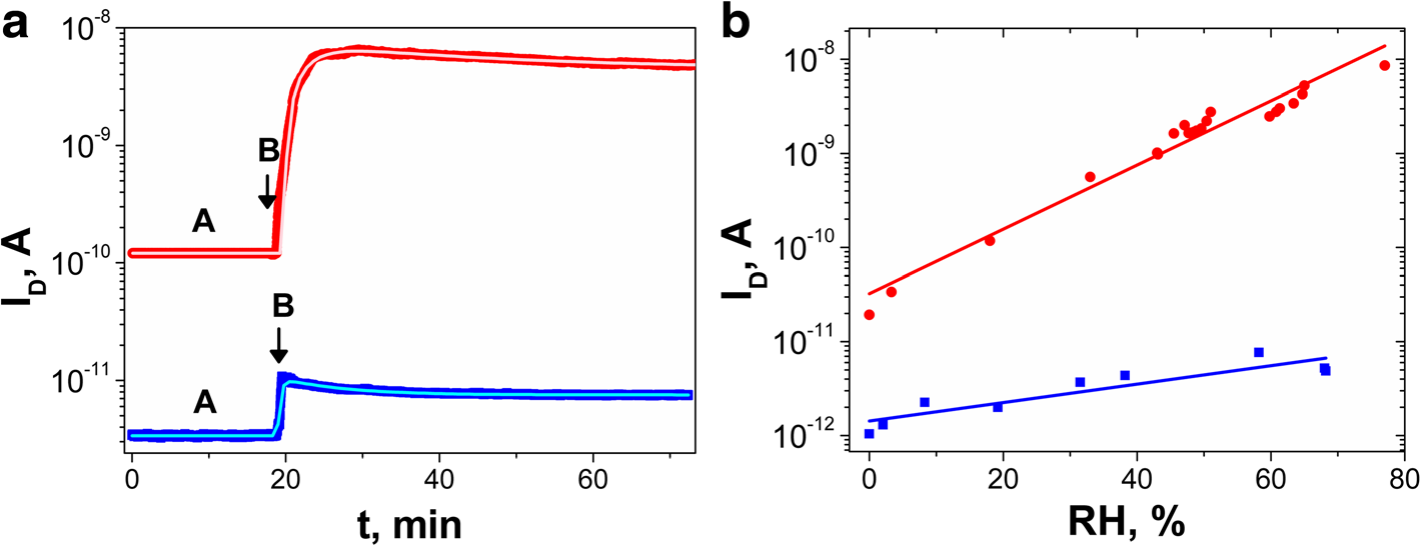
**a** Current responses on step change in humidity from (*A*) RH = 0% to (*B*) RH = 59%. **b** Influence of humidity on electric current flowing through SbSI sensors (*red circle* SbSI gel, *blue square* array of a few SbSI nanowires; *T* = 280 K; *p* = 4 × 10^4^ Pa; *solid lines* represent the best fitted dependences described by Eqs. (1) and (3); values of the fitted parameters are given in Tables 1 and 2)

Figure 2a presents current responses of unilluminated SbSI nanosensors made of gel and an array of a few SbSI nanowires on step change in humidity from RH = 0% to RH = 59%. After the fast increase, the electric currents attain maxima. Then, they decrease slowly to a stationary values. The sensor responses were least square fitted with an empirical relation

1 $$ {I}_D(t)={I}_S+{I}_1{e}^{-\left( t-{t}_{\mathrm{on}}\right)/{\tau}_1}-{I}_2{e}^{-\left( t-{t}_{\mathrm{on}}\right)/{\tau}_2},\kern0.5em \mathrm{f}\mathrm{o}\mathrm{r}\kern0.5em  t\ge {t}_{\mathrm{on}}, $$

where *t*_on_ represents the time when moist N_2_ was injected to the test chamber, *I*_*S*_ is the stationary value of current flowing through sensor exposed to water vapor, *I*_1_ and *I*_2_ are the pre-exponential factors, and *τ*_1_ and *τ*_2_ are the time constants. Values of the fitted parameters are presented in Table 1. It should be underlined that time constants *τ*_1_ and *τ*_2_ determined for array of a few SbSI nanowires are, respectively, 3.1 and 6.4 times smaller than that for SbSI gel.

**Table 1:** Parameters of Eq. (1) fitted to current responses of SbSI nanosensors (Fig. 2a) on humidity step change from RH = 0% to RH = 59% (*T* = 280 K)

Parameter	Sensor	
	Array of a few SbSI nanowires	SbSI gel
*I*_*s*_, A	7.532(4) × 10^−12^	4.51(2) × 10^−9^
*I*_1_, A	3.05(3) × 10^−12^	3.05(2) × 10^−9^
*I*_2_, A	10.4(1) × 10^−12^	7.4(1) × 10^−9^
*τ*_1_, s	466(6)	1430(20)
*τ*_2_, s	25.9(4)	167(1)

Obviously, SbSI gel (Fig. 1a) belongs to a complex system, and its electrical properties can be affected by many factors, e.g., random distribution of contacts between separate nanowires and contacts between nanowires and electrodes. SbSI nanowires aligned in electric field and bonded ultrasonically to Au microelectrodes (Fig. 1c) represent much less complicated system. Firstly, the reliable SbSI nanowire–Au microelectrode bonds were achieved since nanowire ends were embedded into microelectrodes. According to [7], ultrasonic processing has caused 420% increase of DC electric conductance of the junctions between Au microelectrodes and SbSI nanowires. Secondly, connections between separate SbSI nanowires were avoided. It eliminated the random distributed grain-boundary potential barriers in this type of sensor. Therefore, the sensors made of arrays of a few SbSI nanowires ultrasonically bonded with electrodes exhibited smaller time constants (Table 1).

Numerous different mechanisms of water adsorption are known [15]. Electrostatic force dominates the charge compensation and facilitates water adsorption. Both van der Waals and dipole–dipole interactions of water with ferroelectric SbSI could lead to H_2_O adsorption. The increase of electric current flowing through unilluminated SbSI gel and array of a few SbSI single nanowires with increase of humidity can be explained as follows. A proton-transfer process was established in [10] as the dominant conduction mechanism through the adsorbed water on SbSI nanowires. Acceptors in sonochemically prepared SbSI gel exist due to the iodine vacancies in the SbSI lattice [16]. At low humidity, H_2_O molecules are very easily adsorbed chemically on the surface of SbSI by a dissociation mechanism

2 $$ {\mathrm{H}}_2\mathrm{O} + {\mathrm{V}}_{\mathrm{I}} - + {\mathrm{h}}^{+}\to {\mathrm{V}}_{\mathrm{I}}-{\mathrm{OH}}^{-}+{\mathrm{H}}^{+}, $$

where V_I_ denotes the ionized iodine vacancy, V_I_-OH^**−**^ is a hydroxyl group occupying an iodine lattice site, H^+^ represents the free proton, and h^+^ is a hole. According to [17], two mechanisms of the influence of electrical conduction by H^+^ ions generated by this reaction can exist. The one of them relies on the H^+^ ion jumps through interstitials sites and/or their transport through channels in crystalline structure into the interior of grains. Secondly, the H^+^ ions could be transported by jumps between adjacent OH^−^ groups. Such groups are trapped by defects existing in grain boundaries and/or near sample surfaces [17].

The response of SbSI nanosensors at high humidity is determined by superficially adsorbed water vapor. H_2_O molecules can be physisorbed on the SbSI nanowire via hydrogen bonding with increasing RH. The adsorbed water molecule associates with the neighboring H_2_O molecules and forms clusters on nanowires surfaces, as well as at the nanowire boundaries. Then, the nanowire’s conductance increases a lot (Fig. 2) according to Grotthuss’ chain reaction [18], where proton transfer occurs among the hydronium and an ion-conductive layer forms on the surface of the SbSI nanowires.

The large number of contacts between separate nanowires in SbSI gel favors water adsorption. Therefore, the increase of dark current with increase of humidity from dry to wet environment (Fig. 2) is much higher in the case of SbSI gel than in the case of a few, aligned SbSI nanowires. The experimental results presented in Fig. 2b were least square fitted with the following dependence

3 $$ {I}_D\left(\mathrm{RH}\right)={I}_{D0}\ {e}^{\upalpha_{\mathrm{H}} \cdot \mathrm{RH}}, $$

where *I*_*D*_(RH) represents the electric current for relative humidity (RH), *I*_*D*0_ means the pre-exponential factor that describes electric current in dry N_2_ (RH = 0%), and the coefficient α_H_ is related to the sensitivity of electrical conductivity on humidity. The values of the fitted parameters are presented in Table 2.

**Table 2:** Parameters of Eq. (3) fitted to current responses of SbSI nanosensors on humidity (Fig. 2b)

Sensor	α_H_, %^−1^	*I*_*D*0_, pA
Array of a few SbSI nanowires	0.0226(39)	1.43(23)
SbSI gel	0.0787(34)	32.5(54)

Many effects may be responsible for the exponential RH dependence of conductance. For example, it can be linked with the change of permittivity arising from water adsorption [19]. The exponential behavior can also be attributed to the fact that the Debye screening length is much larger than nanowire radius so that the whole nanowire volume is affected by the gating of water molecules on the surface [20]. In another approach [21], exponential dependence on humidity is evoked by jumps over the potential barrier in order to move from one to the next equilibrium. Future investigations of this phenomenon are needed.

Figure 2b shows that in the case of an array of a few SbSI nanowires, the dark current rises only twice with increase of humidity from dry to wet environment (RH = 70%), whereas the *I*_*D*_ for SbSI gel enhances exponentially by nearly three orders of magnitude with the same increase of humidity (Fig. 2b). It demonstrates that the electrical conductance of assemblies of SbSI nanowires in moist N_2_ is mainly caused by H_2_O clusters agglomerated on the nanowire boundaries.

**Fig. 3:**
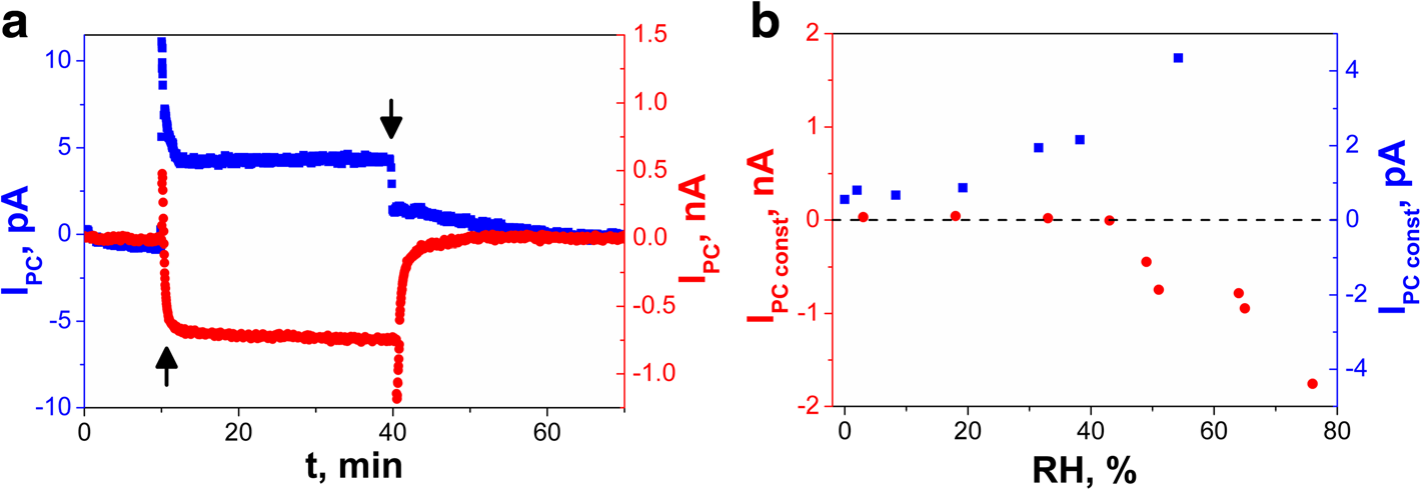
**a** Photoconductivity current responses on switching on (*arrow up*) and switching off (*arrow down*) illumination of (*red square*) SbSI gel and (*blue square*) array of a few SbSI nanowires in moist N_2_ (RH = 53%). **b** Influence of humidity on stationary value of photoconductivity current flowing under constant illumination (*λ* = 488 nm; *T* = 280 K; *p* = 4 × 10^4^ Pa)

Figure 3a shows the comparison of qualitatively different DC photoconductivity current (*I*_PC_) responses on illumination of SbSI gel and array of a few SbSI nanowires in wet nitrogen (RH = 53%). In the first case, the so-called negative photoconductivity is observed. While in the latter case, only positive photoconductivity exists. In both cases after switching on illumination, the electric photoconductivity current increases fast, attains maximum, and then slowly decreases with time to a stationary value. Obviously, the rise of *I*_PC_ after switching on illumination is influenced by photogeneration of excess carriers in the semiconducting SbSI. The first pulse in the transient characteristic exhibits a complex shape which can be recognized as a so-called hook anomaly, observed usually for infrared detectors [22].

Figure 3b shows interesting influence of humidity on value of photoconductivity current *I*_PCconst_ flowing under constant illumination of SbSI nanosensors in N_2_. In the case of few SbSI nanowire array, the final settled value of photocurrent is positive in the whole range of RH. In the case of SbSI gel, the value of photocurrent under constant illumination is positive for small and medium RH, while it is negative for RH over critical value RH_C_ = 39.8% (Fig. 3b). It is quite different from the exponential changes of electric current flowing through the unilluminated SbSI gel (Fig. 2b).

A mechanism behind the negative photoconductivity of Ce_2_O nanowires [23] and Co-doped ZnO nanobelts [24] in ambient air has been recognized as photodesorption of water molecules from surfaces of these nano-objects. Mentioned mechanism seems to be very probable in the investigated case of SbSI gel. The photodesorption of water from SbSI nanowires can involve a photonic or thermal interaction of light with semiconducting SbSI.

**Fig. 4:**
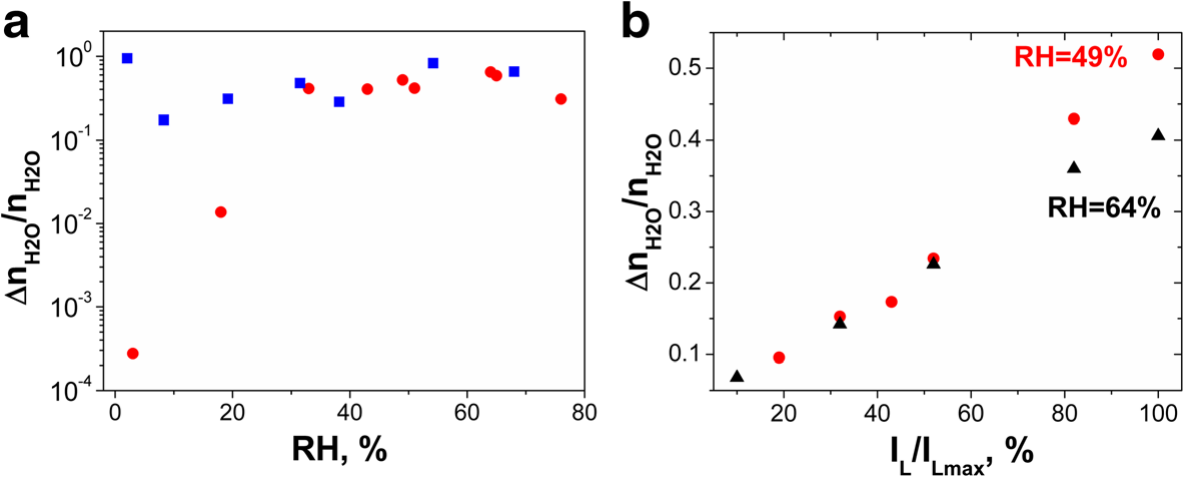
Ratio of desorbed to adsorbed H_2_O molecules as a function of **a** humidity and **b** light intensity for (*red circle*, *black triangle*) SbSI gel and (*blue square*) array of single SbSI nanowires (*λ* = 488 nm; *T* = 280 K; *p* = 4 × 10^4^ Pa; *red circle*—49% RH, *black triangle*—64% RH)

The ratio of desorbed (*Δn*_H2O_) to adsorbed H_2_O molecules (*n*_H2O_) on SbSI surface was calculated using the following relation

4 $$ \frac{\varDelta {n}_{\mathrm{H}2\mathrm{O}}}{n_{\mathrm{H}2\mathrm{O}}}=\frac{I_{\mathrm{PCmax}}-{I}_{\mathrm{PCconst}}}{I_A-{I}_{S0}}, $$

where *I*_*S*0_ represents the bias current flowing through a sample in vacuum, *I*_*A*_ denotes the current enhancement due to additional conductance caused by water adsorption, *I*_PCmax_ is the maximum value of photoconductivity current flowing after switching on illumination, *I*_PCconst_ is the stationary value of photoconductivity current flowing under constant illumination. The influence of humidity on *Δn*_H2O_/*n*_H2O_ ratio is presented in Fig. 4a. This parameter seems to be independent of RH in the case of an array of a few SbSI nanowires. The different behavior is observed for SbSI gel. For small values of RH, water molecules are strongly trapped in multiple nanowire system due to H_2_O adsorption near contacts between SbSI nanowires and near contacts between SbSI nanowires and electrodes. Therefore, the ratio of desorbed to adsorbed H_2_O molecules is small for low humidity. With increasing humidity, the adsorbed water molecules are weakly bonded to SbSI surface in subsequent layers. So, they can be more easily photodesorbed with sensor illumination. The ratio of *Δn*_H2O_/*n*_H2O_ increases about three orders of magnitude with the increase of RH up to 33% and becomes stable for higher RH. Increase of illumination intensity (*I*_*L*_) causes the increase of number of H_2_O molecules desorbed from SbSI gel (Fig. 4b).

**Fig. 5:**
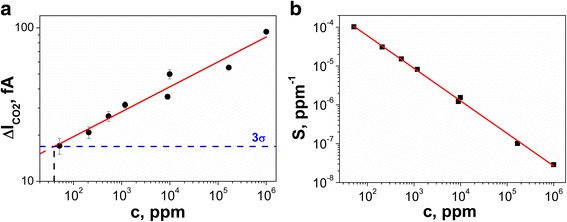
Current response (**a**) and sensitivity (**b**) of a few SbSI nanowires array as a function of CO_2_ concentration (*T* = 304 K, *E* = 1.5 × 10^6^ V/m, *p* = 9.8 × 10^4^ Pa); *red solid lines* represent the best fitted dependences described by Eqs. (5) and (7); values of the fitted parameters are presented in the text; *blue dashed line* represents three times the standard deviation of the noise

Figure 5a presents the influence of CO_2_ concentration on current response of a few SbSI nanowire array. The experimental results were fitted with the following empirical dependence

5 $$ \varDelta {I}_{\mathrm{CO}2}=\varDelta {I}_0\cdot {c}^{\varphi}, $$

where ΔI_CO2_ represents the change in sensor current at a CO_2_ concentration (*c*), Δ*I*_0_ = 9.3(12) fA is the pre-exponential factor, the coefficient *φ* is related to the sensitivity of electrical conductivity on CO_2_, and it equals *φ* = 0.162(15) when concentration is expressed in parts per million units.

The limit of sensor detection is defined as the value of sensor sensitivity that is greater than three times the standard deviation of the noise signal [25]. In the case of experiments with CO_2_, the maximum standard deviation of the noise signal was 5.6 fA. The detection limit of carbon dioxide *c*_min_ = 40(31) ppm determined for array of a few SbSI nanowires is much lower than the CO_2_ detection limit published for FIGARO TGS 4161 (350 ppm [26]) and SnO_2_ sensors (1000 ppm [27]).

According to [28], the definition of current response sensitivity (*S*) is the following:

6 $$ S=\frac{1}{C}\cdot \frac{\varDelta {I}_{\mathrm{CO}2}}{I_0}, $$

where *I*_0_ is the reference value of the sensor exposed to dry nitrogen (without the CO_2_). Combining Eqs. (5) and (6) together, the sensitivity can be expressed finally as

7 $$ S={S}_0\cdot {c}^{\varphi -1}, $$

where *S*_0_ = ΔI_CO2_/*I*_0_. Figure 5b shows the influence of CO_2_ concentration on sensitivity of SbSI nanosensor. The decrease of the sensitivity with increasing gas concentration suggests that with the rise of number of adsorbed CO_2_ molecules, the density of active sites on the SbSI surface is reduced.

Carbon dioxide molecule is usually regarded as an electron acceptor [29–32]. It means that CO_2_ adsorption at a semiconductor surface involves electron transfer from a semiconductor into the CO_2_ molecule. It leads to the formation of a partially charged species CO_2_^δ−^ through interactions with surface atoms [29]. This adsorbate has no longer the linear symmetry of the free CO_2_ molecule [29, 31]. For n-type semiconductor, the electron-accepting adsorbates CO_2_^δ−^ are responsible for the depletion of electrons and decrease of electric conductance [33, 34]. In the case of p-type semiconductor, formation of CO_2_^δ–^ ions enhances number of holes in the valence band, resulting in the increase of electric conductance [33].

Taking into account the results of experiments presented in Fig. 5a, it should be concluded that electric conductivity of the investigated SbSI nanowires is p-type. This conclusion is consistent with XPS analysis of sonochemically prepared SbSI [13, 16] and powdered SbSI crystals [35]. It was revealed elsewhere [7] that adsorption of nitrous oxide causes increase of electric current flowing through SbSI nanosensor, as should be in the case of p-type semiconductor. As to the nature of the conductive carriers in SbSI, it was proposed that iodine vacancies in SbSI lattice play the role of acceptors [36] or that some of the S^2−^ ions that replace the I^−^ ions play the part of acceptors [37].

It is worth mentioning that the working temperature of SbSI sensor is relatively low (e.g., in comparison to metal oxide semiconductor gas sensors [33, 38]). The fact that electrical conductivity is influenced by adsorption does not necessarily mean that chemisorption occurs [39]. Indeed, physisorbed molecules (by polarizing in the process and forming shallow traps for free carriers by their field) may charge the surface and, hence, change the conductivity [39].

## Conclusions

SbSI nanosensors made of xerogel are much better for humidity sensing than the SbSI sensors fabricated as arrays of aligned single nanowires. Probably, the main reason of this behavior is water adsorption in the form of clusters of H_2_O molecules agglomerated on the nanowire-boundaries and near the contacts between nanowires. However, response time of SbSI nanosers made of xerogel is larger than response time of SbSI sensors fabricated as arrays of aligned single nanowires ultrasonically bonded with electrodes.

It should be noted that for the first time influence of humidity and illumination on number of desorbed water molecules from SbSI surface has been analyzed. In the case of low RH the relative desorption of water from SbSI gel is much weaker than from arrays of single nanowires.

To the best of our knowledge, the electrical response of SbSI nanowires affected by CO_2_ adsorption is reported for the first time. SbSI nanosensors made of arrays of aligned single nanowires ultrasonically bonded with electrodes are much better for CO_2_ sensing than the SbSI sensors fabricated as xerogel. They present high performance. The fabricated array of a few SbSI nanowires has exhibited low CO_2_ detection limit of 40(31) ppm. It makes the SbSI sensors competitive to other types of carbon dioxide sensors.

SbSI sensors have shown increase in electric conductance upon exposure to CO_2_, what proves the p-type conductivity of the SbSI nanowires. It is in agreement with literature data for sonochemically produced SbSI in ethanol. The CO_2_ sensing mechanism seems to involve formation of CO_2_^δ–^ species interacting with SbSI surface.

Due to small size, small power consumption, relatively low operating temperature, and negligible heating system for recovery, SbSI nanosensors are attractive for use in many fields such as indoor air quality, early fire detection, and industrial processes. Moreover, the presented fabrication of SbSI nanosensors is cheap and easy to integrate into electronic and control devices.

## Abbreviations

RH: Relative humidity
SbSI: Antimony sulfoiodide
SEM: Scanning electron microscopy
